# Beyond pathways: genetic dissection of tocopherol content in maize kernels by combining linkage and association analyses

**DOI:** 10.1111/pbi.12889

**Published:** 2018-02-20

**Authors:** Hong Wang, Shutu Xu, Yaming Fan, Nannan Liu, Wei Zhan, Haijun Liu, Yingjie Xiao, Kun Li, Qingchun Pan, Wenqiang Li, Min Deng, Jie Liu, Min Jin, Xiaohong Yang, Jiansheng Li, Qing Li, Jianbing Yan

**Affiliations:** ^1^ National Key Laboratory of Crop Genetic Improvement Huazhong Agricultural University Wuhan China; ^2^ National Maize Improvement Center of China MOA Key Lab of Maize Biology Beijing Key Laboratory of Crop Genetic Improvement China Agricultural University Beijing China

**Keywords:** tocopherols, genetic architecture, genome‐wide association study, QTL mapping, maize

## Abstract

Although tocopherols play an important role in plants and animals, the genetic architecture of tocopherol content in maize kernels has remained largely unknown. In this study, linkage and association analyses were conducted to examine the genetic architecture of tocopherol content in maize kernels. Forty‐one unique quantitative trait loci (QTLs) were identified by linkage mapping in six populations of recombinant inbred lines (RILs). In addition, 32 significant loci were detected via genome‐wide association study (GWAS), 18 of which colocalized with the QTLs identified by linkage mapping. Fine mapping of a major QTL validated the accuracy of GWAS and QTL mapping results and suggested a role for nontocopherol pathway genes in the modulation of natural tocopherol variation. We provided genome‐wide evidence that genes involved in fatty acid metabolism, chlorophyll metabolism and chloroplast function may affect natural variation in tocopherols. These findings were confirmed through mutant analysis of a particular gene from the fatty acid pathway. In addition, the favourable alleles for many of the significant SNPs/QTLs represented rare alleles in natural populations. Together, our results revealed many novel genes that are potentially involved in the variation of tocopherol content in maize kernels. Pyramiding of the favourable alleles of the newly elucidated genes and the well‐known tocopherol pathway genes would greatly improve tocopherol content in maize.

## Introduction

Tocopherols are lipid‐soluble compounds occurring in four forms—α, β, γ and δ. Tocopherols and the four forms of tocotrienols are together known as vitamin E, with α‐tocopherol exhibiting the highest vitamin E activity (DellaPenna and Mène‐Saffrané, 2011). In humans, ataxia with vitamin E deficiency (AVED) is directly connected to α‐tocopherol deficiency (Azzi and Stocker, 2000), and immune response is impaired under vitamin E deficiency (Beharka *et al*., 1997). In addition, sufficient vitamin E intake can reduce the risk of chronic diseases such as atherosclerosis, diabetes and cancer (Azzi and Stocker, 2000; Traber *et al*., 2008). In higher plants, tocopherols protect the photosystem from photo‐oxidation damage and keep polyunsaturated fatty acids (PUFAs) from being peroxidated. In the absence of tocopherols, seed longevity is affected and early seedling development is disrupted (Sattler *et al*., 2004). In addition, tocopherol deficiency leads to impaired photoassimilate export from source leaves in maize, potato and tomato (Almeida *et al*., 2016; Hofius *et al*., 2004; Provencher *et al*., 2001). Similar impairment of photoassimilate export has been observed in *Arabidopsis* at low temperature (Maeda *et al*., 2006). Recent studies also suggest a role of tocopherols in the response to stresses, including low temperatures, drought and high light levels (Eugeni‐Piller *et al*., 2014; Loyola *et al*., 2012; Maeda *et al*., 2006, 2008).

**Figure 1:**
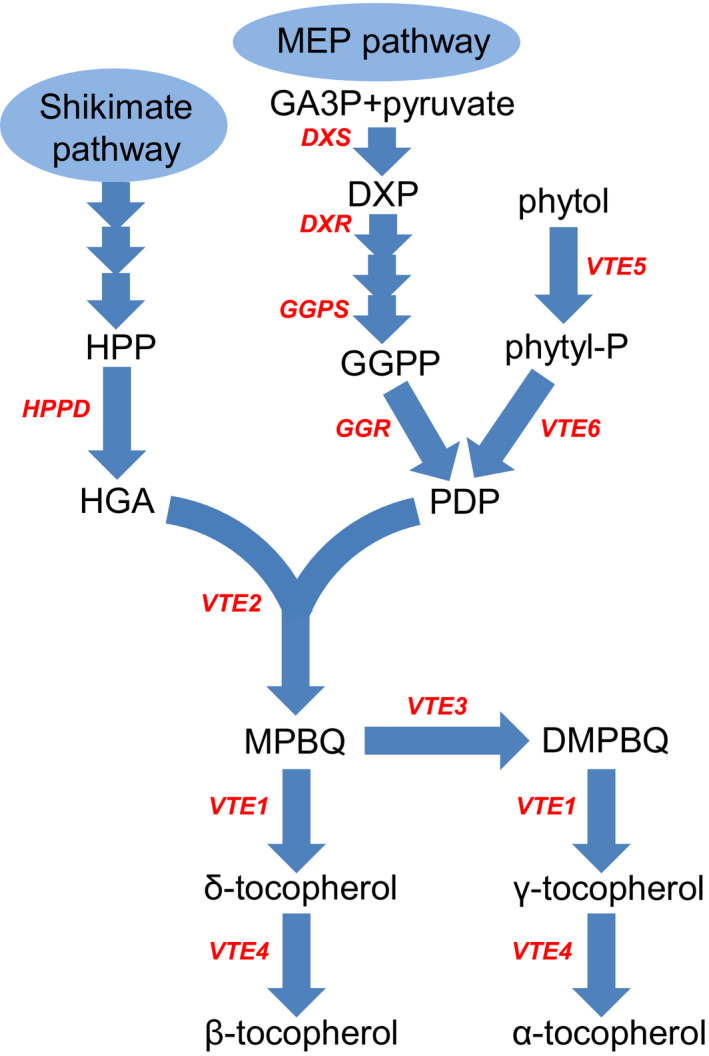
Biosynthetic pathway of tocopherols in higher plants. The abbreviations for compounds in this pathway are as follows: DMPBQ, 2,3‐dimethyl‐5‐phytyl‐1,4‐benzoquinol; DXP, 1‐deoxy‐D‐xylulose 5‐phosphate; GA3P, glyceraldehyde 3‐phosphate; GGPP, geranylgeranyl pyrophosphate; HGA, homogentisic acid; HPP, p‐hydroxyphenylpyruvate; MPBQ, 2‐methyl‐6‐phytyl‐1,4‐benzoquinol; PDP, phytyl diphosphate; Phytyl‐P, phytyl phosphate. Abbreviations for enzymes in the pathway are as follows: DXR, 1‐deoxy‐D‐xylulose‐5‐phosphate reductoisomerase; DXS, 1‐deoxy‐D‐xylulose‐5‐phosphate synthase; GGPS, geranylgeranyl pyrophosphate synthase; GGR, geranylgeranyl reductase; HPPD, p‐hydroxyphenylpyruvate dioxygenase.

The important role of vitamin E in human health and its insufficient intake (https://www.nal.usda.gov/fnic/vitamin-e) (Dror and Allen, 2011) have made biofortification a necessity, especially in developing countries, where sufficient vitamin E sources are often not accessible. Maize (*Zea mays* L.) is considered an ideal crop for tocopherol biofortification due to its wide accessibility and abundant variation in tocopherol content. However, the number of genes that can be used for biofortification is still limited. The biosynthetic pathway of tocopherols has mainly been elucidated in *Synechocystis* and *Arabidopsis* (DellaPenna and Mène‐Saffrané, 2011; DellaPenna and Pogson, 2006; Mène‐Saffrané and DellaPenna, 2010) (Figure 1). Studies in other species, such as tomato, have also greatly enhanced our understanding of the biosynthetic pathways of tocopherols (Lira *et al*., 2016; Quadrana *et al*., 2013). Recent studies suggest that the tocopherol metabolic pathway is not completely understood (Lin *et al*., 2016; Zhang *et al*., 2014), and genes influencing tocopherol content in higher plants continue to be identified (Adato *et al*., 2009; Enfissi *et al*., 2010; Hey *et al*., 2017; Karlova *et al*., 2011; Liao *et al*., 2017; Pellaud *et al*., 2018; Van Eenennaam *et al*., 2004). These findings indicate the complexity of the tocopherol biosynthetic pathway and raise the possibility that there are additional genes that have not been discovered.

Linkage mapping and association mapping are two common and efficient strategies for identifying new genes associated with a certain trait. Both methods have unique advantages. Linkage mapping can detect quantitative trait loci (QTLs) with small effects and low allelic frequencies. Association mapping presents a high mapping resolution that can reach the single‐gene level due to rapid linkage disequilibrium (LD) decay. Both linkage and association mapping rely on natural variations in traits of interest, which are widely observed for tocopherol content among different maize inbred lines (Li *et al*., 2012; Lipka *et al*., 2013), making it feasible to unravel the genetic architecture of tocopherol content through genetic means. Indeed, previous studies have detected QTLs for tocopherols in maize (Chander *et al*., 2008; Wong *et al*., 2003; Xu *et al*., 2012), and the causative polymorphisms were identified (Li *et al*., 2012; Lipka *et al*., 2013). Similarly, in tomato, QTLs for fruit tocopherols have been mapped using an introgression population (Almeida *et al*., 2011). One QTL has been fine‐mapped and cloned to a single gene. Interestingly, epigenetic, rather than genetic, variation underlies this QTL (Quadrana *et al*., 2014). Forty‐one candidate genes involved in tocopherol biosynthesis were identified in the tomato genome, and 16 genes putatively affecting tocopherol content were proposed, including genes that are not directly involved in the tocopherol biosynthetic pathway (Almeida *et al*., 2011). Recently, Diepenbrock *et al*. (2017) identified 14 genes affecting tocochromanol (including tocopherols, tocotrienols and plastochromanol‐8) content, including eight genes from the known tocochromanol biosynthetic pathway and six novel genes. These novel genes are involved in different activities, including two genes encoding chlorophyll biosynthetic enzymes, three genes relating to the transport and storage of lipophilic molecules and one transcription factor. However, the genetic architecture of tocopherol content in maize kernels remains to be elucidated in different genetic backgrounds.

In this study, linkage mapping was carried out in a large collection of six RIL populations, each of which was genotyped with high‐density genetic markers. In combination with GWAS based on high‐density SNPs and transcriptomic data, we attempted to elucidate the genetic architecture of natural tocopherol variation and to identify new genes affecting tocopherol content in maize kernels.

## Results

### Phenotypic variation of tocopherol content in six RIL populations

To determine the genetic architecture of tocopherol content, 11 diverse inbred lines were chosen to develop six RIL populations. These 11 parental lines show abundant diversity for tocopherol content (Figure S1). Five tocopherol‐related traits were measured: γ‐tocopherol content (GT), α‐tocopherol content (AT), δ‐tocopherol content (DT), total tocopherol content (TT) and the α/γ ratio (RT). All tocopherols were measured in each RIL population, which were grown in two or three locations; the one exception was DT in the Dan340/K22 RIL population, which could not be measured accurately. The phenotypes of each RIL population largely followed a normal distribution. The range of phenotypic variation and other statistical data are shown in Table S1. The obtained heritability values were mostly between 0.5 and 0.8. The B73/BY804 population was found to exhibit the highest tocopherol content, and the K22/CI7 population was found to exhibit the highest RT value (Figure S2). These results probably reflect a higher tocopherol content or RT in the parental lines. The diverse genetic backgrounds and the abundant phenotypic diversity provide a basis for detection of new QTLs.

### QTL mapping highlights contributions of both pathway and nonpathway genes to phenotypic diversity

**Figure 2:**
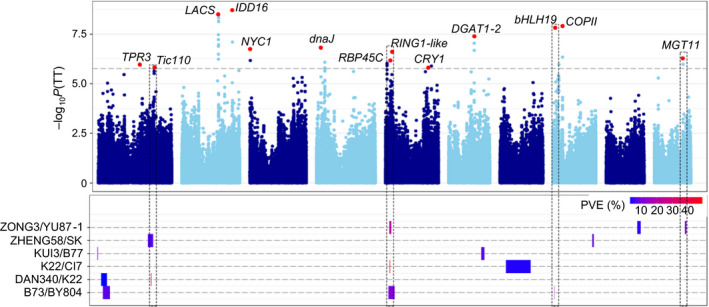
Comparative results between GWAS and QTL analysis for total tocopherol. The upper part shows the GWAS result. The lead independent SNP is shown as a red dot, and the candidate genes are labelled. The lower part shows the QTLs identified from the RIL populations (**Table** S2). The QTL effect is shown as PVE (phenotypic variation explained). Overlapping loci between GWAS and QTL mapping are indicated with dashed rectangles.

In total, 89 QTLs were detected for the five traits, with each QTL explaining 2.9%–48.7% of the observed phenotypic variation (Table 1; Figure 2; Figure S3). The QTL with the largest effect was detected in the K22/CI7 population, showing an *R* ^2^ of 48.7% for TT. Due to the high marker density and high recombination frequency in the RIL populations, 75% of the QTLs were mapped to a physical interval of less than 0.9 Mb, corresponding to the minimal recombination bin in single RIL population. We then combined the QTLs from these six populations, which led to the identification of 41 unique QTLs (Table 1; Table S2), including 14 QTLs that affect two or more traits. The number of QTLs for each trait ranged from 10 to 16, implying that multiple loci control tocopherol content. Among the 41 unique QTLs, 10 have not been identified previously, indicating the advantage of using multiple diverse genetic backgrounds for QTL mapping. Notably, 10 of these 41 unique QTLs represent major QTLs that could each explain more than 10% of the observed phenotypic variation. Six of the major QTLs were detected in multiple populations, and the other four were detected in only one population, suggesting that both common and rare allelic variations underlie the QTLs for tocopherol content.

**Table 1:** Summary of QTLs detected in each RIL population

Traits^a^	QTLs	QTLs in two or more RILs	QTLs in one RIL	Unique QTLs	Unique major QTLs	*R* ^*2*^ (%)	QTL number in each RIL population^b^					
							BB	DK	KC	ZY	ZS	KB
DT	18	2	12	16	3	5.3–13.1	6	/	3	2	4	3
GT	20	3	9	14	4	2.9–46.6	5	3	2	6	2	2
AT	19	3	12	15	4	3.6–35.1	4	3	3	2	2	5
TT	14	3	5	10	3	3.6–48.7	3	2	2	3	2	2
RT	18	4	7	11	3	4.1–40.6	2	3	3	3	3	4
Total	89	15	45	41	10	2.9–48.7	13	6	8	11	9	12

a DT, δ‐tocopherol content; GT, γ‐tocopherol content; AT, α‐tocopherol content; TT, total tocopherol content; RT, α/γ ratio.

b BB, B73/BY804; DK, DAN340/K22; KC, K22/CI7; ZY, ZONG3/YU87‐1; ZS, ZHENG58/SK; KB, KUI3/B77.

To discover whether two QTLs might interact with each other to contribute to phenotypic variation, we performed tests for epistasis between every pair of QTLs in each population (Table S3). The results showed that the interaction between two QTLs could only explain 2.9%–6.5% of the observed phenotypic variation. In addition, no epistatic effect was detected for any tocopherol trait in the ZONG3/YU87‐1 population. These results suggest that epistasis plays a limited role in shaping the natural variation of tocopherol content in maize kernels.

Finally, we examined the colocalization relationship between the QTLs and known genes from the tocopherol pathway. In a recent study, 81 genes involved in tocochromanol biosynthesis were identified in the maize genome (Diepenbrock *et al*., 2017). Among these 81 genes, 49 fell within the confidence intervals of the RIL QTLs (Table S2; Table S4). This vast overlap indicates the indispensable roles of the known tocopherol pathway genes in the six RIL populations examined in this study. However, the possibility cannot be excluded that the corresponding QTLs might be controlled by other genes nearby, as genes involved in the same pathway often appear in clusters (Nützmann and Osbourn, 2014). Moreover, 14 QTLs do not contain genes from the known tocopherol pathway, suggesting a contribution of nonpathway genes to tocopherol phenotypic diversity.

### Fine mapping of a major pleiotropic QTL

**Figure 3:**
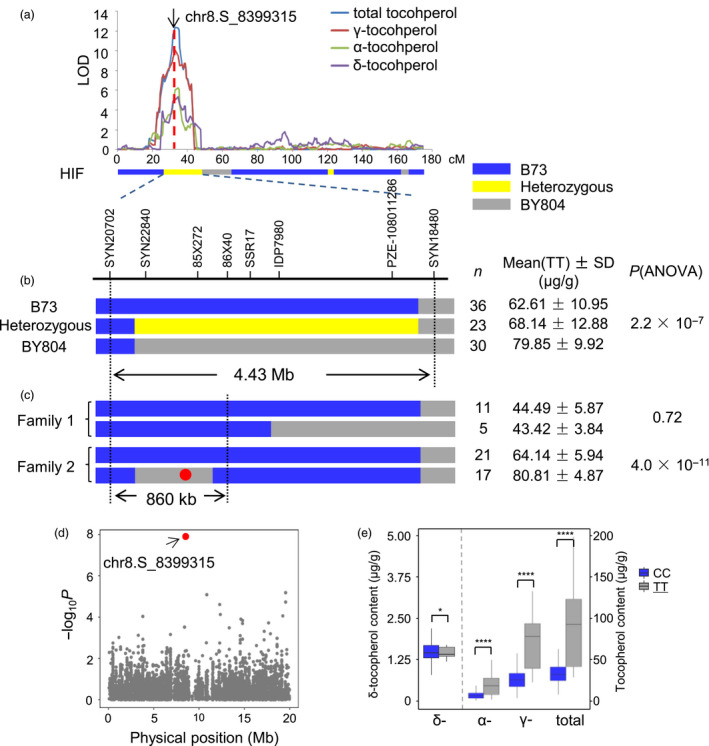
Fine mapping of a major QTL on chromosome 8. (a) A major pleiotropic QTL was detected on chromosome 8 in the B73/BY804 population. A HIF founder heterozygous in the QTL region was chosen for fine mapping. The position of the lead SNP from GWAS is labelled with an arrow. (b) The QTL was validated through the progeny test. Significant differences in total tocopherol contents were detected among the three haplotypes (B73, BY804 and heterozygous) using ANOVA. (c) The QTL was fine‐mapped to an 860‐kb interval. The red dot shows the position of the lead SNP from GWAS. (d) Association of total tocopherol with SNPs in the chromosome 8 region where the QTL was identified. The lead SNP is shown in red. (e) A *t*‐test was performed on the association panel, grouped by the genotype of the lead SNP. The favourable genotype is underlined. *P < 0 .05; ****P < 0.0001.

A major QTL explaining 17.1% of the phenotypic variation for TT was detected in the B73/BY804 population on the short arm of chromosome 8 (Figure 3; Table S2). This QTL also controlled GT and DT. Moreover, according to the QTL mapping results from one environment, AT was also influenced by this QTL (Figure 3a). The recombination bin with the highest logarithm of odds (LOD) value was from 8.42 to 8.56 Mb for TT and from 8.36 to 8.42 Mb for both GT and DT. The confidence intervals for TT, GT and DT were 7.87 to 8.73 Mb, 6.14 to 10.09 Mb and 7.39 to 11.36 Mb, respectively. To validate this QTL and identify the underlying gene, a RIL that was heterozygous at the QTL interval was chosen to develop a heterogeneous inbred family (HIF). One‐way ANOVA revealed significant differences in tocopherol content among the three haplotypes (Figure 3b). This QTL showed an additive effect, with the tocopherol contents of the heterozygous individuals being close to the average contents of the two homozygous parental haplotypes. Recombinants were screened and identified for fine mapping, and the QTL was mapped to an 860‐kb interval containing 30 genes (7.76–8.62 Mb in the B73 reference genome AGPv2) (Figure 3c; Table S5). This region coincided with the peak bin obtained from linkage mapping. Interestingly, one gene from the tocopherol pathway, *DXR2*, was located within this small region. Further fine mapping or other methods, such as candidate gene association studies, will be needed to identify the causative gene underlying the QTL.

### Identification of new loci for tocopherol content in a genome‐wide association study

We performed GWAS with a mixed linear model (MLM) for four tocopherol‐related traits, GT, AT, TT and RT, in an association panel with 508 diverse lines (AMP508). In total, 282 significant SNPs were detected, which were located in or near 86 genes (Table S6; Figure S4). Many of these SNPs are in significant LD. To identify a set of SNPs with independent effects, we conducted a conditional GWAS in which the effect of the most significant SNP was considered as a covariate, thus allowing the detection of SNPs that are not in LD with the covariate SNP. This analysis identified a total of 32 significant SNPs with independent effects (‘lead SNPs’) (Table S7), among which there were 10 SNPs for GT, 8 for AT, 14 for TT and 12 for RT (Table 2). As TT is mostly explained by changes in GT, we observed strong overlap of significant SNPs for these two traits. Although RT is related to both AT and GT, we only observed overlap of significant SNPs between RT and AT. AT is derived from GT via a one‐step enzyme reaction, and the availability of GT does not appear to be a limiting factor for this reaction.

**Table 2:** Summary of significant SNPs detected in GWAS

Traits	No. of significant SNPs	No. of genes with significant SNPs	No. of independent SNPs	Chromosomes of independent SNPs
γ‐tocopherol	43	16	10	1,2,4,5,6,8,10
α‐tocopherol	166	43	8	1,2,3,4,5
Total tocopherol	66	27	14	1,2,3,4,5,6,8,10
α/γ ratio	116	32	12	2,4,5,6,9,10
Total	282	86	32	1,2,3,4,5,6,7,8,9,10

Many of the significant SNPs that were significant in AMP508 colocalized with the QTLs identified in our RIL populations. These included 10 SNPs that were significant for the same traits between the two types of populations and an additional eight loci that were significant for different traits (Table S7). Among the 18 colocalized SNPs, eight segregated in the RIL populations, and the direction of the effect was the same between the RIL population and AMP508; that is, the parent providing the increasing additive effect in the RIL population also harboured the favourable allele over the other parent in the association panel. For the other 10 SNPs, the SNP either did not segregate in the RIL populations (9/10) or showed the opposite effect between the two populations (1/10), suggesting that these SNPs may merely be in LD with the causative sites of the QTLs.

Notably, one significant SNP identified through GWAS (chr8.S_8399315) fell into the QTL that was fine‐mapped to the 860‐kb interval (Figure 3c, d). Student's *t*‐test analysis of AMP508 grouped by this SNP showed extreme significance (Figure 3e), suggesting that this SNP was either the causative site itself or was in close LD with the causative site. The closest gene to this SNP encodes a putative transcription factor (*GRMZM2G066057*) that is not involved in the tocopherol biosynthesis pathway. As a *DXR* homolog is also located within this QTL, it remains elusive which gene is the actual functional gene.

### Frequency distribution of favourable alleles in a natural population

**Figure 4:**
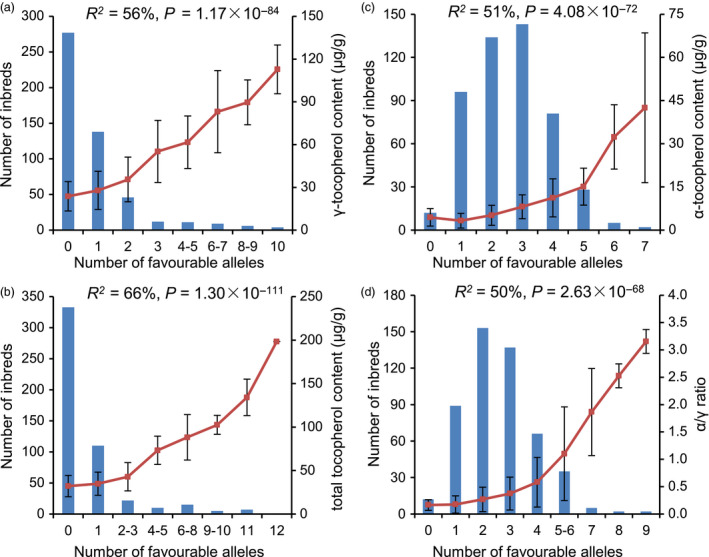
Distribution of the favourable alleles of GWAS lead SNPs in AMP508. The inbred lines from AMP508 are grouped by the number of favourable alleles. The number of lines in each group is shown as a blue bar. The average phenotype of each group is shown as a red square, and the standard deviation is shown as an error bar. The percentage of phenotypic variation that could be explained by the number of favourable alleles is noted for each trait in the plot. The significance level among different groups obtained from ANOVA is also shown.

The favourable allele frequency was less than 10% for most (24) of the 32 significant lead SNPs. To determine whether the favourable alleles of these SNPs came from different lines or were enriched in a few lines, the 508 lines were grouped by the number of favourable alleles they contained for each trait (Figure 4). This analysis revealed two different patterns.

First, more than half of the inbred lines (277/503, GT data were not available for five lines) did not contain any favourable alleles for the 10 loci identified for GT, and the majority of the remaining lines only contained one favourable allele (Figure 4a). A similar pattern was observed for TT (Figure 4b). As expected, the average tocopherol content increased as favourable alleles accumulated: the mean for GT increased 5 times, from 23.70 to 112.80 μg/g, while that for TT increased 6 times, from 32.15 to 198.35 μg/g. The percentages of phenotypic variation explained by the number of favourable alleles were 56% and 66%, respectively, for GT and TT (calculated via one‐way ANOVA). It was noted that there were several lines that harboured almost all of the favourable alleles and therefore exhibited the highest GT and TT, suggesting that pyramiding of favourable alleles can increase the contents of GT and TT.

The second pattern was found for AT and RT. In contrast to GT and TT, the majority of the panel (489/501, AT and RT data were not available for seven lines) exhibited at least one favourable allele for the significant SNPs identified for both AT and RT (Figure 4c and d). In fact, half of the inbred lines contained at least three favourable alleles, representing one‐third (AT) and one‐fourth (RT) of the identified lead SNPs. As expected, the phenotypic value increased as favourable alleles were pyramided: the mean for AT varied 10‐fold, from 4.41 to 42.51 μg/g, and that for RT varied 20‐fold, from 0.16 to 3.15. The percentages of phenotypic variation explained by the number of favourable alleles were 51% and 50% for AT and RT, respectively. Importantly, the favourable alleles of three significant SNPs for AT and RT were common alleles (>0.5); in contrast, the favourable alleles of almost all of the significant SNPs for GT and TT were rare (Table S7), which might explain the wide occurrence of maize inbred lines with three or more favourable alleles for AT and RT compared with zero to one favourable allele for GT and TT.

These results suggested that the accumulation of favourable alleles explains a large proportion of the observed phenotypic diversity in the AMP508 panel. Based on the finding that 24 of the 32 lead SNPs exhibited a minor (and favourable) allele frequency of less than 0.10, it is suggested that rare allelic variation contributes significantly to tocopherol variation.

### Identification of nontocopherol pathway genes in controlling tocopherol content

The residing regions of the GWAS signals were compared with the 81 pathway genes identified by Diepenbrock *et al*. (2017). In contrast to the vast overlap observed in linkage analysis, only six pathway genes fell into the 1‐Mb flanking intervals of the significant lead SNPs according to GWAS. Among these six genes, four were supported by local eQTLs (Fu *et al*., 2013) and by significant correlations between expression and tocopherol content in AMP508 (Table S4). The four genes are as follows: *PDS2*,* MECS2*,* VTE4* and *GRMZM2G036861* (encoding chorismate synthase). The small overlap between GWAS and pathway genes indicates that the genetic diversity leading to tocopherol variation in natural populations may not be contained in biosynthetic pathway genes.

To nominate candidate genes for the 32 lead SNPs, we identified the genes closest to the SNPs as well as all of the genes within the 100‐kb flanking interval (Tables S7 and S8). The results showed that most (27/32) of these SNPs were located within transcribed regions, in addition to two SNPs in promoters and three in intergenic regions (Table S7). These 32 genes closest to the SNPs were involved in diverse functions and gene ontology (GO) analysis did not reveal any significant terms (Figure S5). However, a close examination of the 32 candidate genes revealed three genes involved in chlorophyll metabolism and chloroplast function (Table S7). *GRMZM2G170013* was significant for AT, and its orthologue in *Arabidopsis*,* NYC1*, encodes chloroplastic chlorophyll b reductase, which is involved in chlorophyll b degradation (Jia *et al*., 2015; Sato *et al*., 2009). *GRMZM5G888696* was significant for GT and TT, and its orthologue in *Arabidopsis*,* Tic110*, functions in protein import into the inner envelope membrane of the chloroplast and is essential for plastid biogenesis (Flores‐Pérez *et al*., 2016; Inaba *et al*., 2005; Nakai, 2015). The function of *GRMZM5G888696* was further supported by a pleiotropic QTL that was detected in two independent RIL populations (ZHENG58/SK and DAN340/K22) (Tables S2 and S7). *GRMZM2G024739* was significant for GT and TT, and its orthologue, *CRY1*, has been reported to regulate chlorophyll biosynthesis (Kobayashi and Masuda, 2016; McCormac and Terry, 2002; Stephenson and Terry, 2008).

Furthermore, we found that natural variations in fatty acid pathway genes affect phenotypic variation in tocopherol content, which is supported by at least four points of evidence. The first was that five of the 32 genes affecting tocopherol content in AMP508 also showed a significant association with oil content in the same panel (Table S7), including DGAT, which has recently been shown to affect tocopherol content in *Arabidopsis* (Pellaud *et al*., 2018). The second point of evidence was that the five genes that were significant for both oil and tocopherol were located within tocopherol QTLs, and one gene—*DGAT*—also fell within a QTL for oil content (Yang *et al*., 2010). Third, several QTLs for oil content colocalized with tocopherol QTLs. A previous study identified nine QTLs for oil content in maize kernels using the B73/BY804 RIL population (Yang *et al*., 2010), which was one of the linkage populations included in this study, and five of the nine QTLs coincided with tocopherol QTLs in this population (Table S7). Lastly, a quantitative genome‐wide association study (qGWAS) identified many genes from the fatty acid biosynthesis pathway that affect tocopherol content. qGWAS explores the associations between gene expression and tocopherol content taking kinship into account. In this analysis, the expression of 415 genes in kernels 15 days after pollination (DAP) was found to affect tocopherol content. Among these 415 genes, 81 were located within the QTL intervals, and GO analysis revealed significant enrichment in fatty acid biosynthesis (Figure S6).

### Validation of a fatty acid pathway gene in controlling tocopherol content

**Figure 5:**
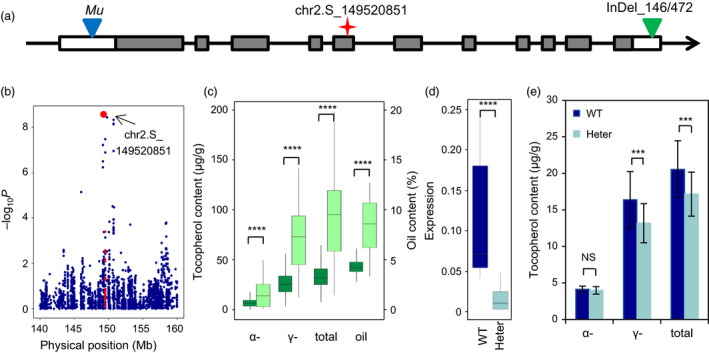
The fatty acid pathway gene *LACS* affects tocopherol content. (a) Gene structure of *LACS*. The red asterisks show the position of the lead SNP detected in GWAS. The blue triangle shows the position of the *Mu* insertion, and the green triangle shows the InDel identified in a previous study (Li *et al*., 2013). (b) Regional association plot of total tocopherol content in AMP508. The red dots represent SNPs within *LACS*. (c) Student's *t*‐test for tocopherol traits and oil content grouped by the *LACS* InDel. The dark green boxes show the haplotype with the 146‐bp deletion and 472‐bp insertion, while the light green boxes show the haplotype with the 146‐bp insertion and 472‐bp deletion. (d) The *Mu* insertion affects *LACS* expression. The expression abundance of the *LACS* gene was normalized based on *Actin‐1*. (e) The *Mu* insertion in *LACS* affects tocopherol content (*n* = 22 for WT and *n* = 32 for Heterozygote). NS, not significant; ***P < 0.001; ****P < 0.0001.

To validate the hypothesis that the fatty acid pathway affects tocopherol content, we focused on one gene, *LACS* (*GRMZM2G079236*). This gene encodes a long‐chain acyl‐coenzyme A (CoA) synthase. Its orthologue in *Arabidopsis* functions to activate fatty acids to acyl‐CoA and participates in multiple processes involved in fatty acid metabolism, including phospholipid, triacylglycerol, and jasmonate biosynthesis and fatty acid β‐oxidation (Fulda *et al*., 2002; Li‐Beisson *et al*., 2013; Shockey *et al*., 2002). A SNP in the 5th exon of *LACS* leading to a synonymous change was significant for AT, TT and GT (Figure 5a and b). Interestingly, this gene was also shown to be responsible for variation of oil content in the same association panel (Li *et al*., 2013). The two tightly linked InDels in the 3′UTR of this gene that affect oil content were significantly associated with TT, GT and AT as well (Figure 5a and c), and the InDels were in LD with the SNP affecting tocopherol content identified in this study (*r* ^2^ = 0.92).

To further confirm the effect of *LACS* on tocopherol content, a mutant of this gene was obtained from the *UniformMu* collection. The *Mu* transposon was inserted in the 5′UTR of *LACS* (Figure 5a), causing a reduction in the expression of *LACS* (Figure 5d). An attempt to achieve homozygous *Mu* insertion failed, indicating that the function of this gene is essential for kernel development. We therefore compared the tocopherol content between the individuals with the heterozygous and wild‐type haplotypes. Compared with the wild‐type individuals, the individuals that were heterozygous for the *Mu* insertion showed significant decreases in GT and TT, which were the same two traits that showed associations in the GWAS (Figure 5a and e). Although *LACS* affects AT in the natural populations, the difference for AT was not statistically significant between the mutant and the wild type (Figure 5e). These findings suggest that *LACS* has a positive effect on tocopherol content.

## Discussion

In this study, linkage and association analyses were combined to detect the genetic architecture of a relatively simple quantitative trait, tocopherol content, for which the metabolic pathway is fairly well understood. The integration of dense genetic markers and a large sample size led to the identification of many QTLs/SNPs that were not identified in previous studies. In total, 32 independent SNPs and 41 unique QTLs (representing 282 SNPs and 89 QTLs) were identified for one or more tocopherol traits in the association and RIL populations, respectively. This represents a significant improvement over the number of significant SNPs/QTLs identified in previous GWAS (18 SNPs by Li *et al*., 2012; 28 SNPs by Lipka *et al*., 2013) or QTL mapping studies (31 QTLs by Chander *et al*., 2008; 30 QTLs by Xu *et al*., 2012). By comparing the results of linkage and association analyses, loci that were shared as well as unique to each analysis were identified. The presence of method‐specific significant loci may have several causes, such as that (i) the parental lines employed to develop the RIL populations may not capture all of the genetic variations present in the association panel, leading to an inability to assess the effects of some loci in the QTL analysis; (ii) some QTLs that are only detected in the RIL populations may be caused by rare genotypic variations for which GWAS shows insufficient detection power; and (iii) the functional SNPs may not segregate in the RIL population, even though the lead SNP in LD does. The lead SNP may be significant in the GWAS because it is in partial LD with the functional SNP but does not cause a phenotypic change in the RIL population.

Another finding was that not all genes in the tocopherol biosynthetic pathway showed significant signals in our GWAS and QTL analyses, suggesting a limited contribution of known tocopherol pathway genes to the observed phenotypic diversity of tocopherol content, as proposed in a recent study (Diepenbrock *et al*., 2017). One possible reason for this is that functional mutations of the pathway genes—especially those involved in rate‐limiting steps—may lead to defects in development or environmental adaptation and will therefore be eliminated by either natural or artificial selection. Alternatively, genetic variations in some pathway genes may not have a phenotypic effect, or the effect may be too small to be detected. On the other hand, many genes that do not directly participate in the tocopherol pathway were shown to significantly affect tocopherol content. Many of these genes were involved in the fatty acid biosynthetic pathway. A relationship between tocopherol and fatty acid metabolism has been reported in previous studies. In tomato, Almeida *et al*. (2016) found that a mutation in *VTE5*, a gene involved in chlorophyll breakdown and phytol recycling, results in reduced accumulation of tocopherols in both the leaves and fruits. Interestingly, lipid metabolism is also affected in the *vte5* mutant, as evidenced by changes in both the abundance and composition of fatty acid phytyl esters (FAPEs), a transient plastidial sink for the deposition of fatty acids and phytol (Gaude *et al*., 2007). In leaf, the level of FAPEs containing linoleic acid (18:2) is increased at least twofold in the *vte5* mutant compared with that in the wild type (Almeida *et al*., 2016). Similarly, Maeda *et al*. (2008) reported that *Arabidopsis* harbouring mutations in *VTE2*, the first gene committed to the tocopherol biosynthetic pathway, exhibited a distinct fatty acid composition from the wild type at low temperature. These studies supported the idea that genes involved in tocopherol biosynthetic pathways either have a direct or an indirect effect on fatty acid metabolism. It remains to be determined whether the opposite is true; that is, the genes from the fatty acid pathway affect tocopherol metabolism. In this study, we found that natural variations in five genes from the fatty acid pathway were associated with tocopherol variation (Table S7). In addition, a mutation in *LACS*, a gene from the fatty acid metabolism pathway, resulted in an altered tocopherol profile (Figure 5). Moreover, the influence of *DGAT*, another fatty acid pathway gene that was found to affect tocopherol content in this study, was recently shown to affect tocopherol content in *Arabidopsis* (Pellaud *et al*., 2018). Together, these results suggest the existence of complicated cross‐talk between fatty acid and tocopherol metabolic pathways.

Our results also suggested that genes involved in plastid function and chlorophyll metabolism affect natural variations in tocopherol content (Table S7). In fact, the phytol derived from chlorophyll degradation has been proposed to be the primary source of the prenyl chain for tocopherol biosynthesis in *Arabidopsis* seeds (Valentin *et al*., 2006). Several genes from the chlorophyll metabolic pathway have been shown to affect tocopherol content (Figure S7). In tomato, *PPH* (encoding a phytol hydrolytic enzyme) silencing lines exhibit impaired chlorophyll breakdown and an altered tocopherol content (Guyer *et al*., 2014; Lira *et al*., 2016). In pepper fruit, a QTL controlling chlorophyll content was shown to have altered tocopherol content (Brand *et al*., 2012). In *Arabidopsis*, changes in the expression of a gene encoding chlorophyll synthase affect tocopherol levels (Zhang *et al*., 2015). Similarly, *vte5* (encoding phytol kinase) and *vte6* (encoding phytyl‐phosphate kinase) mutants exhibit reduced tocopherol content (Dorp *et al*., 2015; Valentin *et al*., 2006). Most recently, two *POR* genes (encoding protochlorophyllide reductase) involved in chlorophyll biosynthesis were found to contribute to phenotypic variation in maize kernels (Diepenbrock *et al*., 2017). In this study, three genes involved in chloroplast function were identified to affect tocopherol content (Table S7). Together, these results suggest that natural variations in genes involved in chlorophyll metabolic pathways and plastid function play an important role in shaping phenotypic variation in tocopherols in maize kernels.

Our study sheds light on the genetic architecture of tocopherols in maize kernels. Tocopherol content is controlled by a few loci with major effects and a number of loci with minor effects, in contrast to the patterns for several other complex quantitative traits in the same genetic background (Liu *et al*., 2017; Pan *et al*., 2017; Xiao *et al*., 2016). Epistasis only plays a minor role, as reported by others (Liu *et al*., 2017; Pan *et al*., 2017; Xiao *et al*., 2016). In terms of the allele frequency of the identified loci, minor alleles with a frequency of <0.1 were the favourable alleles for most of the significant loci identified. The low occurrence of favourable alleles in natural populations is consistent with the observation that many modern maize inbred lines contain no or very few favourable alleles. We found that increasing the number of favourable alleles led to an almost linear increase in tocopherol content. The additive effects of many loci with rare favourable alleles in maize populations suggest that biofortification of tocopherols (vitamin E) via pyramiding of superior alleles would be a useful strategy that would be applicable in plant breeding.

## Materials and methods

### Association panel, genotyping and association analysis

The AMP508 association mapping panel consists of 508 diverse inbred lines, including temperate and tropical/subtropical elite inbred lines, inbred lines derived from landraces collected from China and abroad that have been selfed for many generations, and 35 high‐oil lines (Yang *et al*., 2011). The panel was genotyped using the Illumina MaizeSNP50 BeadChip, containing 56 110 SNPs. RNA was extracted from kernels 15 days after pollination in 368 lines and used to profile gene expression on the Illumina platform (Fu *et al*., 2013). In total, approximately 1.03 million high‐quality SNPs were obtained, with 560K SNPs exhibiting a minor allele frequency (MAF) ≥0.05. The expression level of 28 769 genes was determined from the RNA‐seq data. The genotype of unsequenced lines was imputed based on the MaizeSNP50 genotypes using a two‐step method (Yang *et al*., 2014).

Genome‐wide association analysis was conducted using an MLM implemented in TASSEL3.0 (Bradbury *et al*., 2007; Yu *et al*., 2006; Zhang *et al*., 2010). The MLM identified significant associations between polymorphisms and phenotypes while controlling for population structure and kinship (Li *et al*., 2012). The significance threshold was set as *P *<* *1.8 × 10^−6^ (*P *<* *1/*n*,* n* = total marker number) based on Bonferroni correction of multiple testing. To distinguish independent functional sites and significant sites in LD, conditional GWAS was performed using the most significant SNP within the 2‐Mb flanking regions as a covariate. All independent significant loci were then employed as covariates to identify potential new significant loci. The genes within 100‐kb upstream and 100‐kb downstream of the lead SNP were extracted from the B73 reference genome (AGPv2).

### Linkage populations, genotyping and QTL mapping

Six RIL populations, B73/BY804, DAN340/K22, K22/CI7, ZONG3/YU87‐1, ZHENG58/SK and KUI3/B77, that were developed previously (Chander *et al*., 2008; Xiao *et al*., 2016; Xu *et al*., 2012) were genotyped using the Illumina MaizeSNP50 BeadChip. High‐density genetic maps of each RIL population were constructed (Pan *et al*., 2016). Each population contains approximately 200 lines, and the number of unique recombination events ranged between 2100 and 3071.

Composite interval mapping was performed using WinQTLCart2.5 (Wang *et al*., 2012; Zeng, 1994) with a scanning interval of 0.5 cM and a window size of 10 cM. ‘Model 6’ in the ‘Zmapqtl’ module was employed, and forward and backward stepwise regression was performed with five markers for background control. The QTL cut‐off was set as an LOD > 3.0. The two‐LOD descending interval from the QTL peak was defined as the QTL confidence interval. If two or more QTLs exhibited overlapping confidence intervals with peaks less than 20 Mb apart, they were combined and designated one unique QTL.

### Field trials and tocopherol content measurement

The AMP508 panel was planted in 2009 at three locations in China, Yunnan, Sichuan and Hainan, with two replicates per location (Li *et al*., 2012). All lines were self‐pollinated, and kernels from the middle of at least three ears were employed to extract and measure tocopherols. The extraction procedures were adapted from previous studies (Chander *et al*., 2008; Kurilich and Juvik, 1999). Tocopherol quantification was performed via high‐performance liquid chromatography (HPLC) (Li *et al*., 2012). Three tocopherol components, DT, GT and AT, were measured. The total tocopherol content was obtained by summing the three forms of tocopherol. The ratio of AT to GT was also calculated. The best linear unbiased predictor (BLUP) was employed to obtain the final phenotypic value to be used for association analysis. Because the DT content of more than one‐third of the lines in the panel was too low to be quantified precisely, DT was not used as a target trait in GWAS.

The six RIL populations were planted in Yunnan and Chongqing in 2011, with one random‐block replication per location. The B73/BY804 population was also planted at third location, in Hainan. The lines were self‐pollinated, and tocopherols were extracted as for the AMP508. The tocopherol measurement for these six RIL populations employed a procedure that is slightly different from the one employed for the AMP508. Specifically, tocopherols were measured with an ultra‐performance liquid chromatography (UPLC) from the Waters Corporation (Milford, MA), using a reverse‐phase BEH C18 column (1.7 μm particle, 2.1 × 100 mm). The mobile phase consisted of acetonitrile:methanol (75:25 v/v) containing 0.05% triethylamine (TEA) and 0.0028% butylated hydroxytoluene (BHT). The flow rate was 0.3 mL/min. The DT, GT and AT standards came from the Sigma‐Aldrich Company (St. Louis, MO). Both the standards and samples were dissolved in the mobile phase. The photo‐diode array (PDA) detector was used at 295 nm for absorbance detection. Three forms of tocopherols could be separated within 5 min on the UPLC. The detection time for each sample was 10 min, to reduce interference between samples. Through UPLC analysis, DT was quantified in five of the six RIL populations, with the exception of the DAN340/K22 RILs. The BLUP value calculated for each trait in each RIL population was used for QTL mapping (Table S1).

### Detection of epistatic QTLs

For every tocopherol trait, additive‐by‐additive epistatic interactions were measured for all QTL pairs detected for that trait within each RIL population. Two‐way ANOVA was employed for this analysis (*P *<* *0.05) (Yu *et al*., 1997). The epistatic effect was derived by comparing the residual of the model containing both single‐locus effects and two‐locus interaction effects with that of the model containing only single‐locus effects.

### Fine mapping using HIFs

Residual heterozygosity occurs widely in RIL populations (McMullen *et al*., 2009) and is an important resource for the fine mapping of QTLs. In this study, an RIL heterozygous for a major QTL on chromosome 8 was chosen to develop HIFs. A homozygous background was achieved via molecular marker‐assisted selection based on the high‐density genetic map. Molecular markers were developed for the QTL based on the reference genome and were then employed to screen for recombinants within the QTL. The identified recombinants were selfed, and their progeny were used for phenotypic measurements. The method employed for tocopherol measurement was the same as that used for the RIL populations.

### Gene Ontology analysis

GO enrichment analysis was conducted using agriGO (http://bioinfo.cau.edu.cn/agriGO/) (Du *et al*., 2010). Singular enrichment analysis mode was employed, with ‘Zea mays ssp V5a’ as the supported species, and the ‘suggested background’ of this version of the maize genome was chosen as the reference. A false discovery rate (FDR) of 0.05 was used to identify significant GO terms.

### *Mu* transposon insertion mutant

A mutant in which a *Mu* transposon was inserted at the 5′UTR of the *LACS* gene was found in MaizeGDB (https://www.maizegdb.org, line number: UFMu‐05576) and ordered from the Maize Genetics Cooperation Stock Center. The genotype was identified via PCR amplification with three primers: a primer pair that specifically amplifies the genomic sequence flanking the *Mu* insertion in *LACS* and the primer TIR6, which is specific to the terminal inverted repeat sequences on both sides of the *Mu* transposon. The primer sequences were as follows (5′ → 3′):

Forward: AAGTAGCCACGGGGTTATGC
Reverse: GGAGTAGTCGGAGCCGTTTC
TIR6: AGAGAAGCCAACGCCAWCGCCTCYATTTCGTC

### Expression analysis of a *LACS* mutant

RNA samples of ear leaves were obtained from both the heterozygous mutant and the wild type during flowering. RNA was extracted using the HUAYUEYANG RNA Extraction Kit and reverse‐transcribed into cDNA. Quantitative PCR was conducted using Vazyme AceQ SYBR Green Master Mix. The expression level of the *LACS* gene was normalized based on *Actin‐1*. The primer sequences employed for these assays were as follows (5′ → 3′):

LACS‐Forward: ACCGGAGAGGATGATATGGGATAC
LACS‐Reverse: GCTCCAGCACAGGTTTCAGTCAAA
Actin1‐Forward: TACGAGATGCCTGATGGTCAGGTCA
Actin1‐Reverse: TGGAGTTGTACGTGGCCTCATGGAC

### qGWAS

To estimate the relationship between tocopherol content and gene expression, as well as the co‐expression pattern between two genes, a linear mixed model (LMM) was adopted, adding relatedness between lines as a covariate. The cut‐off was set as *P *<* *1/*n* (*n* = total number of genes detected) (Wen *et al*., 2016) for co‐expression between genes. For associations between tocopherol and gene expression, the top 100 genes with the lowest *P* value for each trait were used. Considering the main role of *ZmVTE4* in determining α‐tocopherol content and the α/γ ratio, the association of the two traits was re‐calculated to identify new genes after controlling for *ZmVTE4* as a covariate.

### Statistical estimates

BLUP values of tocopherol content were calculated using the MIXED procedure of SAS software (SAS Institute Inc.). As only one replicate of each RIL population was planted in each location, phenotypic variance (*V*_p_) was divided into genotypic variance (*V*_g_), environmental variance (*V*_en_) and error (*V*_er_). Heritability was calculated as *V*_g_/*V*_p_ according to Holland *et al*. (2003).

## Conflict of interest

The authors declare no conflict of interests.

## Author contributions

J.Y. and Q.L. designed the research; H.W., S.X, Y.F., N.L, W.Z., K.L. and Q.L. conducted the experiments; H.W., S.X., H.L., Y.X., Q.P., M.D., J.L. and M.J. analysed and visualized the results; W.L., Q.L. and X.Y. provided field and material support; and H.W., Q.L. and J.Y. wrote the manuscript.

## Supporting information


**Figure S1** Phenotypic distribution of tocopherol contents in the 11 parents of the six RIL populations.
**Figure S2** Box‐plot showing the distribution of tocopherol‐related traits in the six RIL populations.
**Figure S3** Comparative results between GWAS and QTL analysis for three tocopherol traits.
**Figure S4** Quantile‐quantile plots of GWAS results.
**Figure S5** GO analysis of the 32 genes closest to the 32 lead SNPs from GWAS.
**Figure S6** GO enrichment of significant genes in qGWAS.
**Figure S7** Chlorophyll metabolism genes that potentially affect tocopherols in higher plants.

**Table S1** Statistical analysis for tocopherol traits in six RIL populations.
**Table S2** QTL information mapped in each RIL population.
**Table S3** Percent variation explained by epistasis between pairs of QTLs.
**Table S4** Eighty‐one *a priori* candidate genes identified in a previous study.
**Table S5** Candidate genes in the QTL region on chromosome 8.
**Table S6** All significant SNPs for tocopherol traits detected by GWAS.
**Table S7** Independent SNPs from conditional GWAS.
**Table S8** List of genes within the 100 kb flanking intervals of lead SNPs identified via GWAS.
