# Supplementary material for: Beyond pathways: genetic dissection of tocopherol content in maize kernels by combining linkage and association analyses
Source: Plant Biotechnol J. 2018 Feb 20;16(8):1464–75. doi: 10.1111/pbi.12889 (PMC6041443; doi:10.1111/pbi.12889)
Supplement: Supplementary file 1 — Figure S1 Phenotypic distribution of tocopherol contents in the 11 parents of the six RIL populations. Figure S2 Box‐plot showing the distribution of tocopherol‐related traits in the six RIL populations. Figure S3 Comparative results between GWAS and QTL analysis for three tocopherol traits. Figure S4 Quantile‐quantile plots of GWAS results. Figure S5 GO analysis of the 32 genes closest to the 32 lead SNPs from GWAS. Figure S6 GO enrichment of significant genes in qGWAS. Figure S7 Chlorophyll metabolism genes that potentially affect tocopherols in higher plants. [file PBI-16-1464-s002.pptx]

## Slide 1
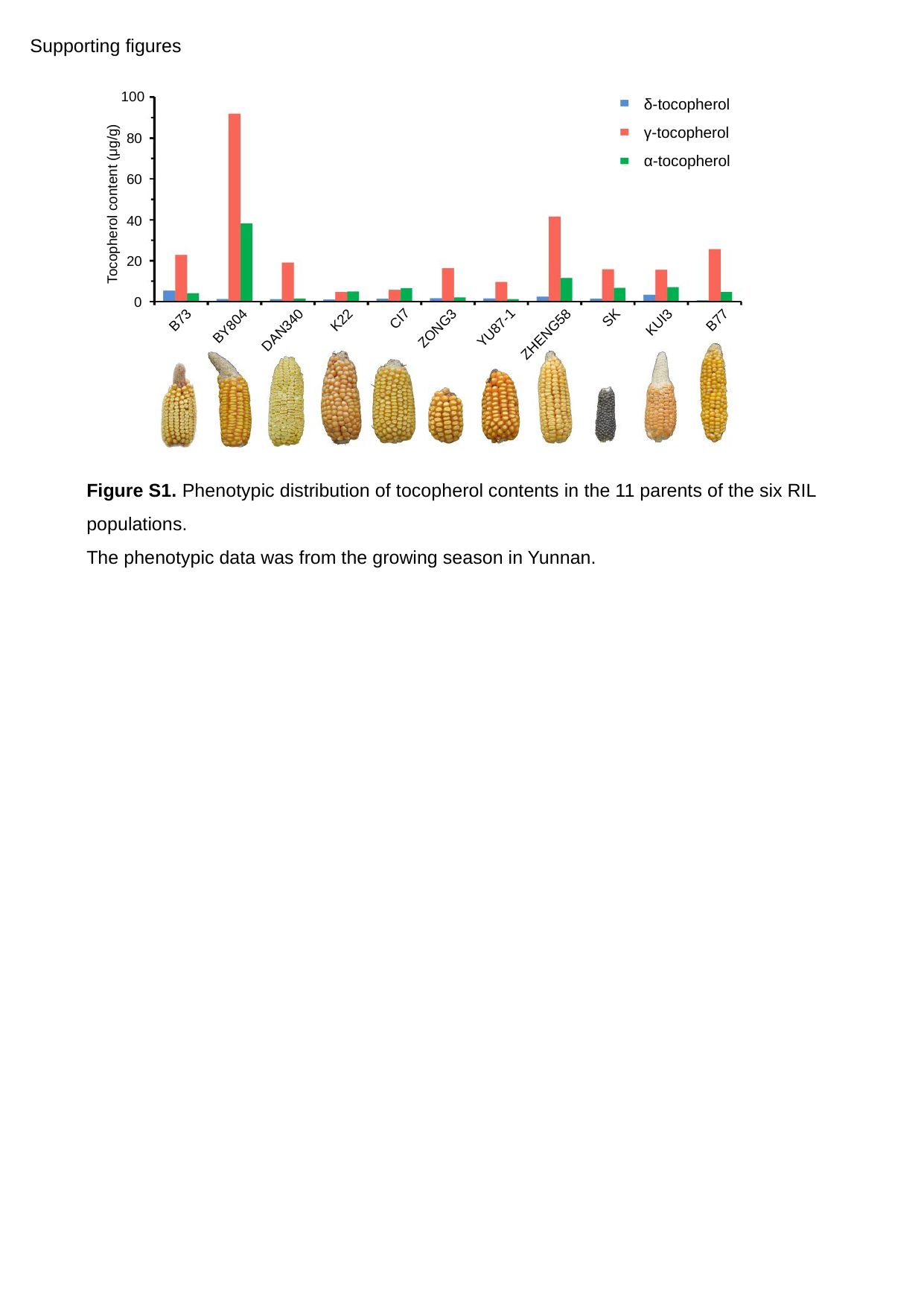

Supporting figures
100
δ-tocopherol
γ-tocopherol
α-tocopherol
80
60
Tocopherol content (μg/g)
40
20
0
B73
BY804
DAN340
K22
CI7
ZONG3
YU87-1
ZHENG58
SK
KUI3
B77
Figure S1. Phenotypic distribution of tocopherol contents in the 11 parents of the six RIL populations.
The phenotypic data was from the growing season in Yunnan.

## Slide 2
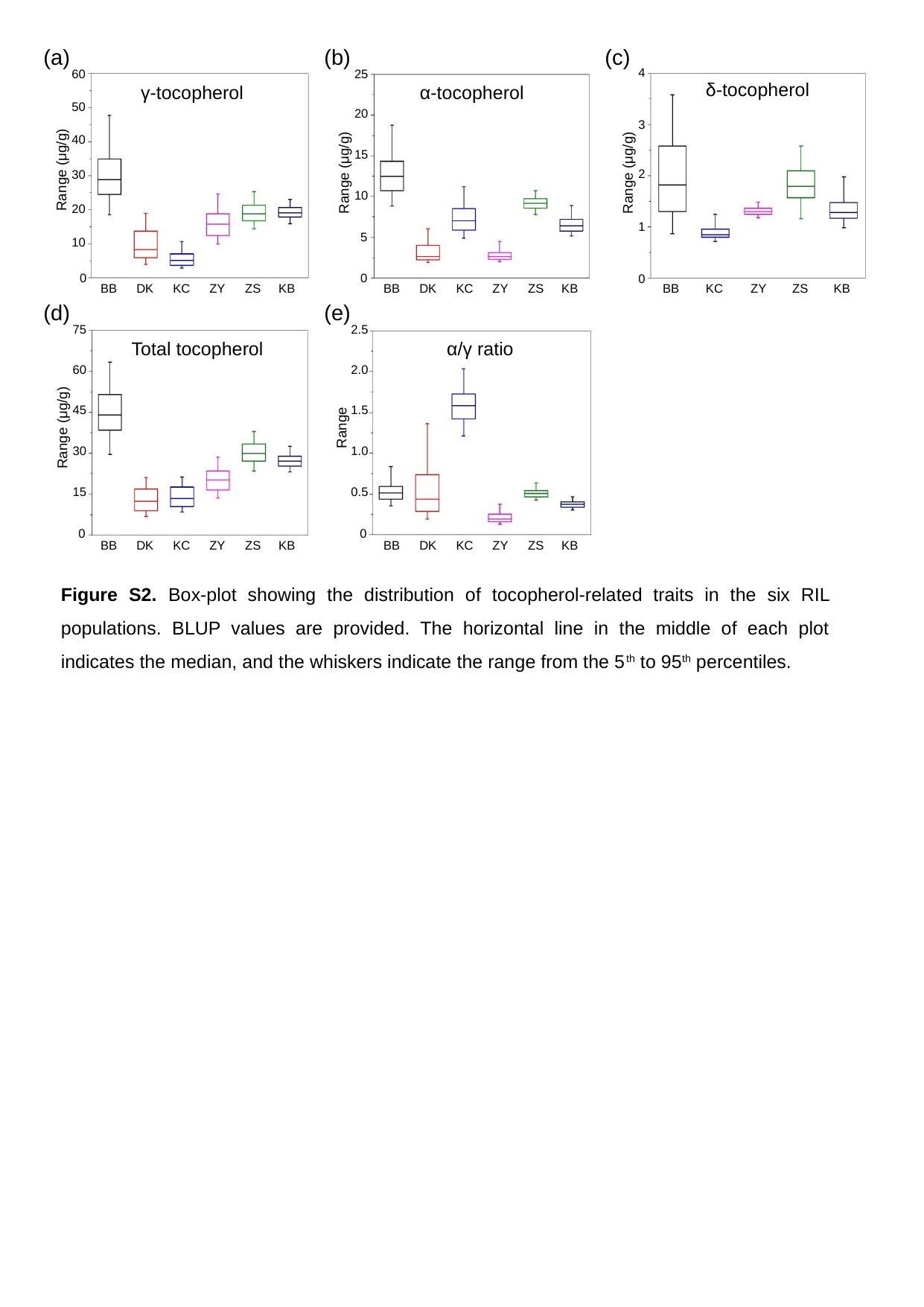

(a)
(b)
(c)
4
25
60
δ-tocopherol
γ-tocopherol
α-tocopherol
50
20
3
40
15
Range (μg/g)
Range (μg/g)
Range (μg/g)
2
30
10
20
1
5
10
0
0
0
ZS
ZS
DK
KC
ZY
KB
DK
KC
ZY
KB
BB
BB
BB
KC
ZY
ZS
KB
(e)
(d)
75
2.5
Total tocopherol
α/γ ratio
60
2.0
45
1.5
Range (μg/g)
Range
30
1.0
15
0.5
0
0
ZS
ZS
DK
KC
ZY
KB
DK
KC
ZY
KB
BB
BB
Figure S2. Box-plot showing the distribution of tocopherol-related traits in the six RIL populations. BLUP values are provided. The horizontal line in the middle of each plot indicates the median, and the whiskers indicate the range from the 5th to 95th percentiles.

## Slide 3
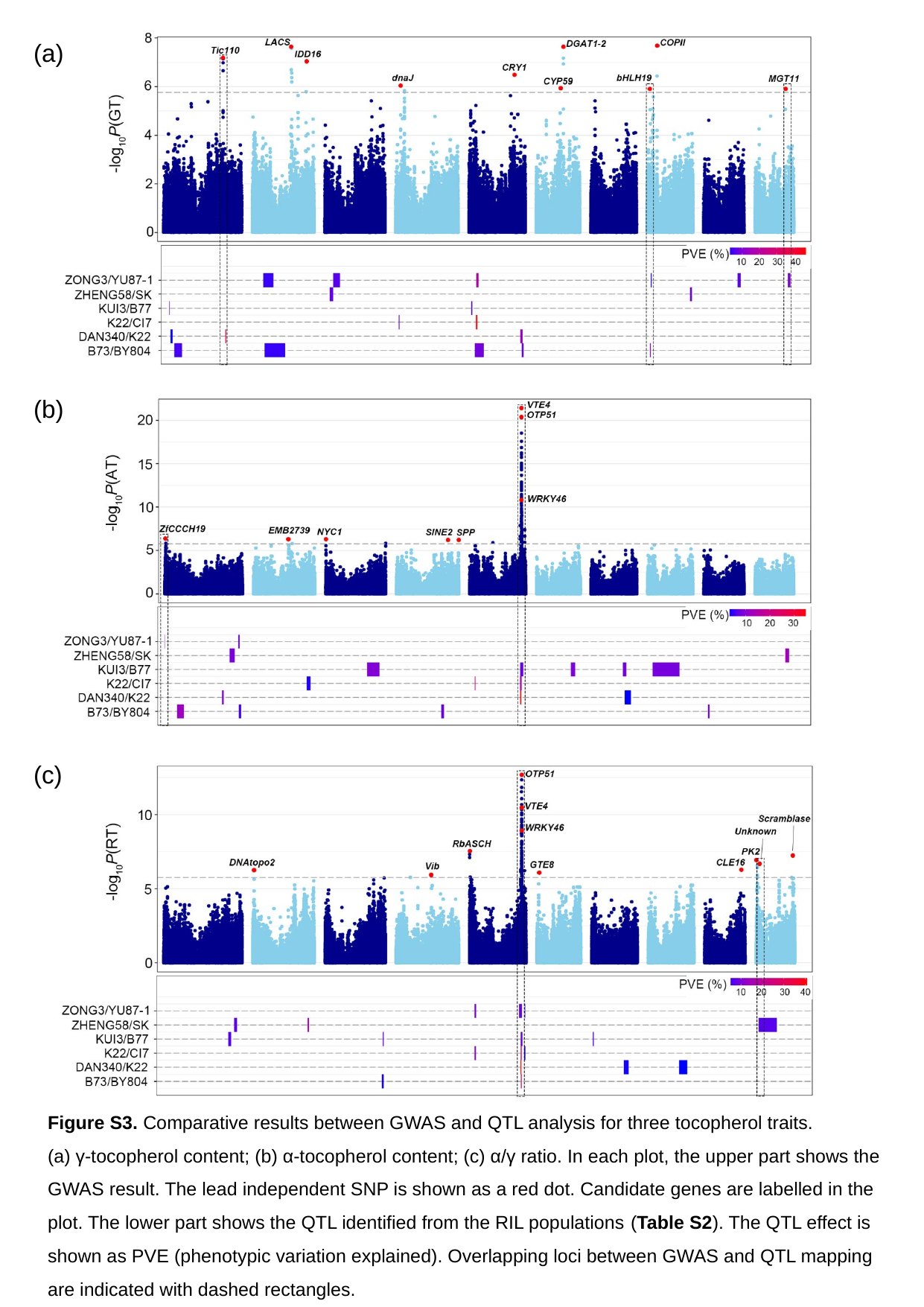

(a)
(b)
(c)
Figure S3. Comparative results between GWAS and QTL analysis for three tocopherol traits.
(a) γ-tocopherol content; (b) α-tocopherol content; (c) α/γ ratio. In each plot, the upper part shows the GWAS result. The lead independent SNP is shown as a red dot. Candidate genes are labelled in the plot. The lower part shows the QTL identified from the RIL populations (Table S2). The QTL effect is shown as PVE (phenotypic variation explained). Overlapping loci between GWAS and QTL mapping are indicated with dashed rectangles.

## Slide 4
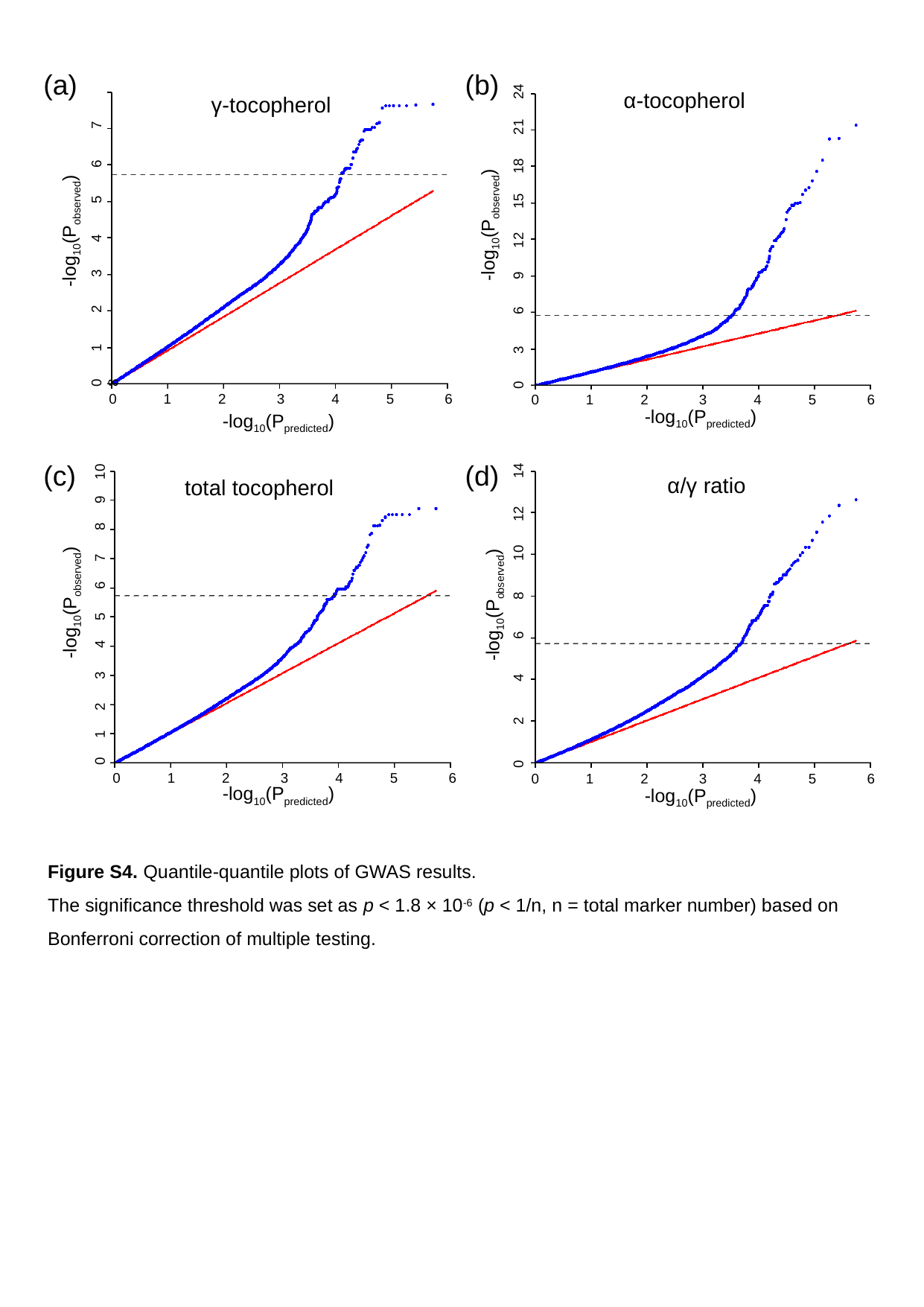

(a)
(b)
α-tocopherol
γ-tocopherol
-log10(Pobserved)
-log10(Pobserved)
0 3 6 9 12 15 18 21 24
0 1 2 3 4 5 6 7 8
0 1 2 3 4 5 6
0 1 2 3 4 5 6
-log10(Ppredicted)
-log10(Ppredicted)
(c)
(d)
α/γ ratio
total tocopherol
0 2 4 6 8 10 12 14
-log10(Pobserved)
-log10(Pobserved)
0 1 2 3 4 5 6 7 8 9 10
0 1 2 3 4 5 6
0 1 2 3 4 5 6
-log10(Ppredicted)
-log10(Ppredicted)
Figure S4. Quantile-quantile plots of GWAS results.
The significance threshold was set as p < 1.8 × 10^-6^ (p < 1/n, n = total marker number) based on Bonferroni correction of multiple testing.

## Slide 5
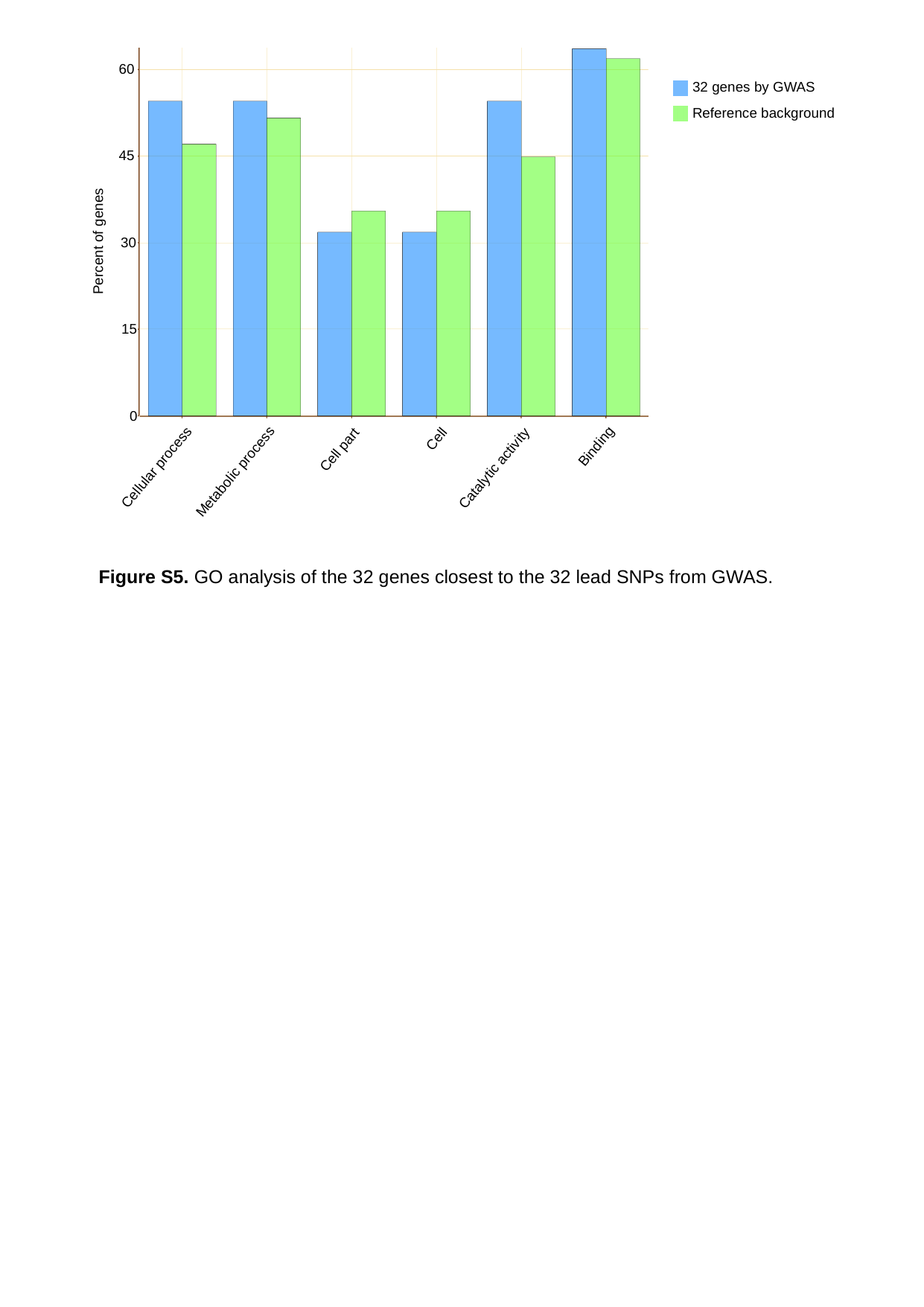

60
32 genes by GWAS
Reference background
45
Percent of genes
30
15
0
Cellular process
Metabolic process
Binding
Cell part
Cell
Catalytic activity
Figure S5. GO analysis of the 32 genes closest to the 32 lead SNPs from GWAS.

## Slide 6
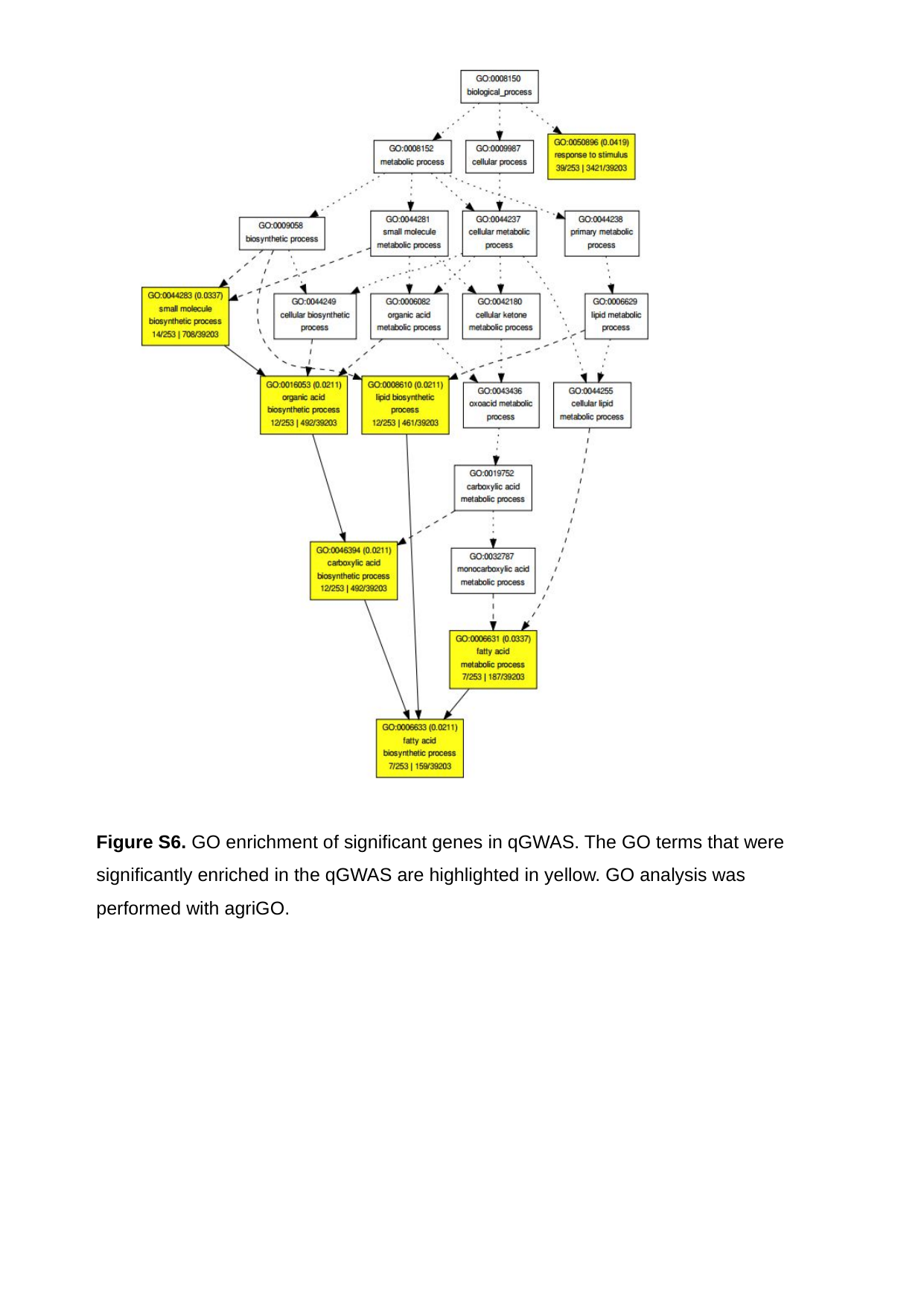

Figure S6. GO enrichment of significant genes in qGWAS. The GO terms that were significantly enriched in the qGWAS are highlighted in yellow. GO analysis was performed with agriGO.

## Slide 7
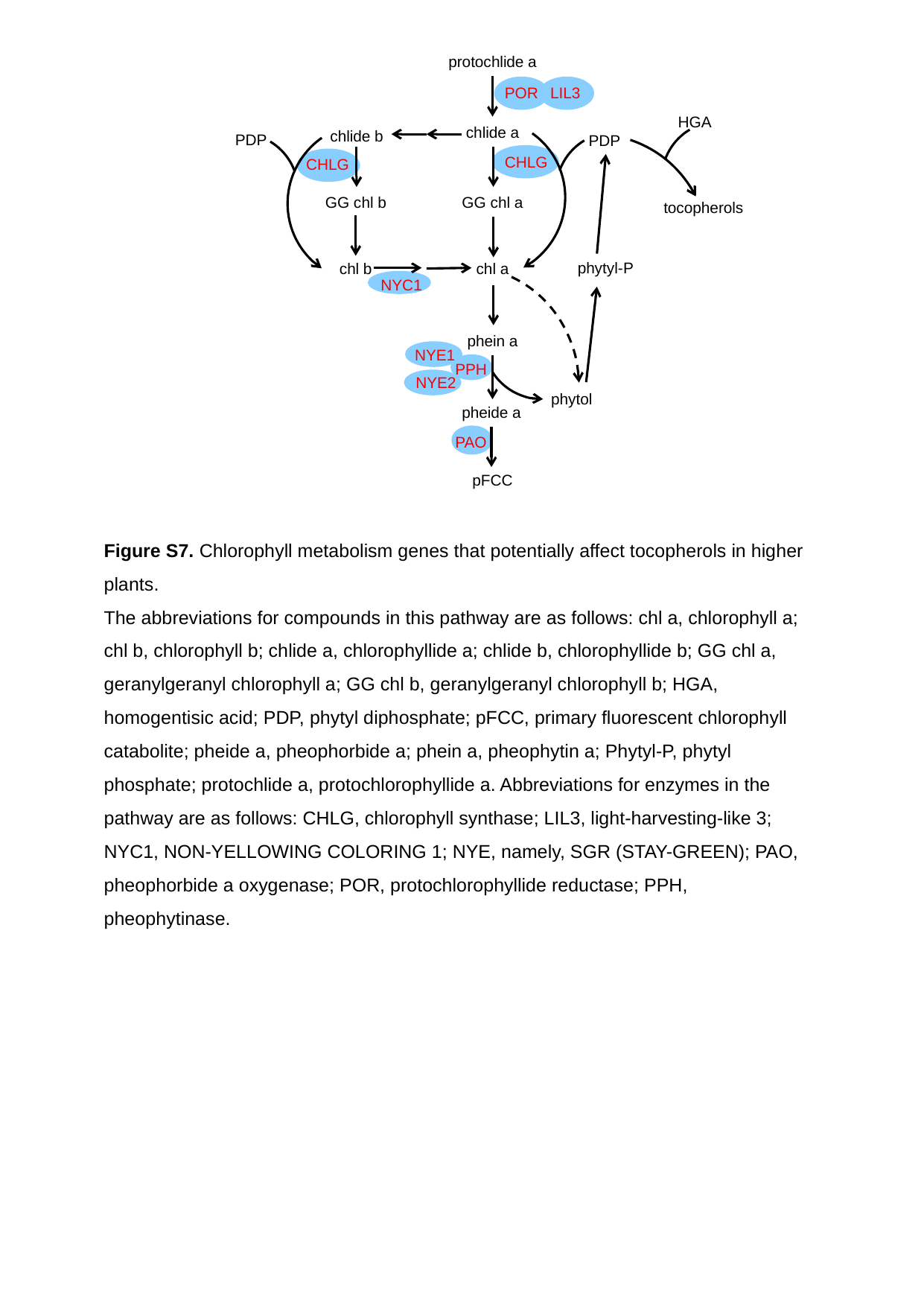

protochlide a
LIL3
POR
HGA
chlide a
chlide b
PDP
PDP
CHLG
CHLG
GG chl b
GG chl a
tocopherols
phytyl-P
chl b
chl a
NYC1
phein a
NYE1
PPH
NYE2
phytol
pheide a
PAO
pFCC
Figure S7. Chlorophyll metabolism genes that potentially affect tocopherols in higher plants.
The abbreviations for compounds in this pathway are as follows: chl a, chlorophyll a; chl b, chlorophyll b; chlide a, chlorophyllide a; chlide b, chlorophyllide b; GG chl a, geranylgeranyl chlorophyll a; GG chl b, geranylgeranyl chlorophyll b; HGA, homogentisic acid; PDP, phytyl diphosphate; pFCC, primary fluorescent chlorophyll catabolite; pheide a, pheophorbide a; phein a, pheophytin a; Phytyl-P, phytyl phosphate; protochlide a, protochlorophyllide a. Abbreviations for enzymes in the pathway are as follows: CHLG, chlorophyll synthase; LIL3, light-harvesting-like 3; NYC1, NON-YELLOWING COLORING 1; NYE, namely, SGR (STAY-GREEN); PAO, pheophorbide a oxygenase; POR, protochlorophyllide reductase; PPH, pheophytinase.
